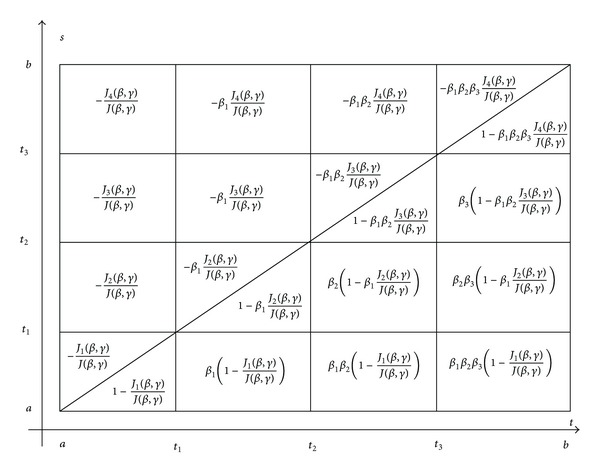# About Positivity of Green's Functions for Nonlocal Boundary Value Problems with Impulsive Delay Equations

**DOI:** 10.1155/2014/978519

**Published:** 2014-02-13

**Authors:** Alexander Domoshnitsky, Irina Volinsky

**Affiliations:** Department of Mathematics and Computer Science, Ariel University, Ariel, Israel

## Abstract

The impulsive delay differential equation is considered (*Lx*)(*t*) = *x*′(*t*) + ∑_*i*=1_^*m*^ *p*_*i*_(*t*)*x*(*t* − *τ*_*i*_(*t*)) = *f*(*t*),  *t* ∈ [*a*, *b*], *x*(*t*_*j*_) = *β*_*j*_ *x*(*t*_*j*_ − 0),  *j* = 1,…, *k*,  *a* = *t*_0_ < *t*_1_ < *t*_2_ < ⋯<*t*_*k*_ < *t*_*k*+1_ = *b*,  *x*(*ζ*) = 0,  *ζ* ∉ [*a*, *b*], with nonlocal boundary condition *lx* = ∫_*a*_^*b*^ *φ*(*s*)*x*′(*s*)*ds* + *θx*(*a*) = *c*, *φ* ∈ *L*_*∞*_[*a*, *b*]; *θ*,  *c* ∈ *R*. Various results on existence and uniqueness of solutions and on positivity/negativity of the Green's functions for this equation are obtained.

## 1. Introduction

Mathematical models with impulsive differential equations attract the topic attention of many researchers (see [1–7]); many important results on boundary value problems and stability of these equations have been obtained. One of possible approaches to study impulsive equations is the theory of generalized differential equations allowing researchers to consider systems with continuous solutions as well as systems with discontinuous solutions and discrete systems in the frame of the same theory [8–15]. In this paper we use the approach proposed in the monograph [1] and developed in [16–20].

Various comparison theorems for solutions of the Cauchy and periodic problems for ordinary differential equations with impulses have been obtained in [17, 21–24]. On the basis of the comparison theorems, tests of stability are proposed in [25, 26]. Theory of impulsive differential equations and inclusions was presented in the book [27].

Equations with nonlocal boundary conditions are applied in modelling different processes (see, e.g., the recent work of Skubachevskii [28]). Nonlocal problems for nonimpulsive functional differential equations were considered in ([25], Chapter 15). Existence results for nonlocal boundary value problems with impulsive equations are presented in [29–35]. There are almost no results on sign constancy of Green's function for impulsive boundary value problems. Concerning nonlocal impulsive boundary value problems, we know, there are no results about positivity/negativity of Green's function. In this paper we propose results of this sort.

In this paper we consider the following impulsive equation:

(1) (Lx)(t)=x′(t)+∑i=1mpi(t)x(t−τi(t))=f(t),                  t∈[a,b],

(2) $$\begin{array}{c} x\left(t_{j}\right)=\beta_{j}x\left(t_{j}-0\right),\;j=1,\ldots ,k, \end{array}$$

(3) $$\begin{array}{c} a=t_{0}<t_{1}<t_{2}<\cdots <t_{k}<t_{k+1}=b, \end{array}$$

(4) $$\begin{array}{c} x\left(\zeta \right)=0,\;\zeta \notin \left[a,b\right], \end{array}$$

with different types of boundary conditions:

(5) lx=∫abφ(s)x′(s)ds+θx(a)=c, φ∈L∞[a,b];                   θ,c∈R,  θ≠0,

(6) $$\begin{array}{c} x\left(a\right)=c, \end{array}$$

(7) $$\begin{array}{c} x\left(a\right)=\gamma x\left(b\right),\;\gamma \in R,\;\;\gamma \neq 1. \end{array}$$

We develop the ideas presented in [17] and we have obtained various results on the existence and uniqueness of solutions for impulsive boundary value problems. The main contribution of the presented paper is the formulation and proof of positivity/negativity conditions for Green's functions for the following impulsive functional differential boundary value problems: (1), (2), (4), and (5); (1), (2), (4), and (6); (1), (2), (4), (7).

## 2. Solution's Representation Formulas

Define the space *D*(*t*_1_,…, *t*_*k*_) of piecewise continuous functions *x* : [*a*, *b*] → *R*, isomorphic to the topological product *L* × *R*, where *L* is the space of measurable essential bounded functions *z* : [*a*, *b*] → *R*, by the following equality:

(8) x(t)=∫atΩ(t,s)z(s)ds+ω(t)α,ω(t)=∑i=1k+1χ[ti−1,ti)(t)∏j=1iβi−j,Ω(t,s)=∑i=1k+1χ[ti−1,ti)(t)χ[ti−1,ti)(s)β0 +∑i=2k+1 ∑r=1i−1χ[ti−1,ti)(t)χ[ti−1,ti)(s)∏j=1i−rβi−j,

where *a* = *t*_0_ < *t*_1_ < *t*_2_ < ⋯<*t*_*k*_ < *t*_*k*+1_ = *b*.

It is clear that *x*(*t*) is absolutely continuous in (*t*_*i*−1_, *t*_*i*_), *i* = 1,…, *k* + 1, satisfying the equality *x*(*t*_*i*_) = *β*_*i*_ *x*(*t*_*i*_ − 0). It is clear that *x*(*t*) has discontinuity of the first kind and is continuous from the right at points *t*_*i*_, *i* = 1,…, *k*.

By [17], the general solution of (1)–(4) has the following representation:

(9) $$\begin{array}{c} x\left(t\right)=C\left(t,a\right)x\left(a\right)+\int_{a}^{t}C\left(t,s\right)f\left(s\right)ds.‍ \end{array}$$

**Theorem 1**

If the boundary value problem (1)–(5) is uniquely solvable in the space *D*(*t*_1_,…, *t*_*k*_) for every essential bounded *f* and *c* ∈ *R*, then its solution can be represented in the form

(10) x(t) =∫abG(t,s)f(s)ds+C(t,a)cθ+∫abφ(s)Cs′(s,a)ds,

where Green's function *G*(*t*, *s*) of this problem is

(11) G(t,s) =C(t,s)−C(t,a)∫sbφ(w)Cw′(w,s)dw+φ(s)θ+∫abφ(w)Cw′(w,a)dw

and *C*(*t*, *s*) = 0  for *t* < *s*.

**Proof**

From the boundary condition (5), we obtain

(12) ∫abφ(s)x′(s)ds+θx(a)=c  ⟹x(a)=cθ−1θ∫abφ(s)x′(s)ds.

Substituting (12) into (9), we obtain

(13) x(t)=C(t,a)θ(c−∫abφ(s)x′(s)ds) +∫atC(t,s)f(s)ds.

The Cauchy function *C*(*t*, *s*) can be represented in the following form:

(14) C(t,s)=∑i=0k ∑j=0kCij(t,s)[Hti(t)−Hti+1(t)]   ×[Htj(s)−Htj+1(s)],

where *C*_*ij*_(*t*, *s*) = *C*(*t*, *s*) in the rectangle *t*_*i*_ ≤ *t* < *t*_*i*+1_, *s*_*i*_ ≤ *s* < *s*_*i*+1_.

Let us take derivative in (9):

(15) x′(t)=Ct′(t,a)x(a)+ddt(∫atC(t,s)f(s)ds)=Ct′(t,a)x(a)+∫atCt′(t,s)f(s)ds +C(t,t)f(t).

Since, according to the definition of the Cauchy function, *C*(*t*, *t*) = 1, we obtain

(16) $$\begin{array}{c} x^{\prime }\left(t\right)=C_{t}^{\prime }\left(t,a\right)x\left(a\right)+\int_{a}^{t}C_{t}^{\prime }\left(t,s\right)f\left(s\right)ds+f\left(t\right). \end{array}$$

Let us substitute (16) into (13) as follows:

(17) x(t) =C(t,a)θ ×(c−∫abφ(w)[Cw′(w,a)x(a)           +∫awCw′(w,τ)f(τ)dτ+f(w)]dw) +∫atC(t,s)f(s)ds=cθC(t,a)−C(t,a)θ ×∫abφ(w)[Cw′(w,a)x(a)        +∫awCw′(w,τ)f(τ)dτ+f(w)]dw +∫atC(t,s)f(s)ds=cθC(t,a)+∫atC(t,s)f(s)ds   −C(t,a)θ∫abφ(w)Cw′(w,a)x(a)dw −C(t,a)θ∫abφ(w)(∫awCw′(w,τ)f(τ)dτ)dw −C(t,a)θ∫abφ(w)f(w)dw.

Since *C*(*t*, *s*) = 0, *s* > *t*, it follows that ∫_*a*_^*t*^ *C*(*t*, *s*)*ds* = ∫_*a*_^*t*^ *C*(*t*, *s*)*ds* + ∫_*t*_^*b*^ *C*(*t*, *s*)*ds* = ∫_*a*_^*b*^ *C*(*t*, *s*)*ds*.

Changing the order of integration in the third double integral, we get

(18) x(t)=cθC(t,a)+∫abC(t,w)f(w)dw −C(t,a)θ∫abφ(w)Cw′(w,a)x(a)dw −C(t,a)θ∫ab∫wbφ(s)Cs′(s,w)f(w)dsdw −C(t,a)θ∫abφ(w)f(w)dw=cθC(t,a) +∫ab[C(t,w)f(w)−C(t,a)θφ(w)Cw′(w,a)x(a)    −C(t,a)θ∫wbφ(s)Cs′(s,w)f(w)ds    −C(t,a)θφ(w)f(w)]dw=cθC(t,a) +∫ab[[C(t,w)−C(t,a)θ      ×(∫wbφ(s)Cs′(s,w)ds+φ(w))]f(w)]dw −∫abC(t,a)θφ(w)Cw′(w,a)x(a)dw=∫ab[C(t,w)−C(t,a)θ   ×(∫wbφ(s)Cs′(s,w)ds+φ(w))]f(w)dw   +C(t,a)θ(c−x(a)∫abφ(w)Cw′(w,a)dw).

Substituting now (16) into (12), we obtain

(19) x(a)=cθ−1θ∫abφ(s) ×[Cs′(s,a)x(a)+∫asCs′(s,w)f(w)dw+f(s)]ds=cθ−x(a)1θ∫abφ(s)Cs′(s,a)ds −1θ∫abφ(s)[∫asCs′(s,w)f(w)dw+f(s)]ds,x(a)[1θ(θ+∫abφ(s)Cs′(s,a)ds)] =1θ(c−∫abφ(s)[∫asCs′(s,w)f(w)dw+f(s)]ds),x(a)=c−∫abφ(s)[∫asCs′(s,w)f(w)dw+f(s)]dsθ+∫abφ(s)Cs′(s,a)ds=−∫abφ(s)f(s)dsθ+∫abφ(s)Cs′(s,a)ds +c−∫abφ(s)[∫asCs′(s,w)f(w)dw]dsθ+∫abφ(s)Cs′(s,a)ds=−∫abφ(s)f(s)ds+∫abf(w)∫wbφ(s)Cs′(s,w)ds dwθ+∫abφ(s)Cs′(s,a)ds +cθ+∫abφ(s)Cs′(s,a)ds.

Substituting (19) into (18), we obtain

(20) x(t)=∫ab[C(t,w)−C(t,a)θ   ×(∫wbφ(s)Cs′(s,w)ds+φ(w))]f(w)dw  +C(t,a)θ(c+[(∫abφ(s)f(s)ds+∫abf(w)             ×∫wbφ(s)Cs′(s,w)ds dw−c)            ×(θ+∫abφ(s)Cs′(s,a)ds)−1]        ×∫abφ(w)Cw′(w,a)dw),x(t)=∫ab[C(t,w)−C(t,a)θ(∫wbφ(s)Cs′(s,w)ds+φ(w))   +(C(t,a)/θ)∫abφ(s)Cs′(s,a)dsθ+∫abφ(s)Cs′(s,a)ds   ×[φ(w)+∫wbφ(s)Cs′(s,w)ds]]f(w)dw  +C(t,a)θ(c−c∫abφ(s)Cs′(s,a)dsθ+∫abφ(s)Cs′(s,a)ds)=∫abG(t,w)f(w)dw+g(t).

It is clear that Green's function *G*(*t*, *s*) of this problem is of the following form:

(21) G(t,s)=C(t,s)−C(t,a)θ(∫sbφ(τ)Cτ′(τ,s)dτ+φ(s)) +(C(t,a)/θ)∫abφ(w)Cw′(w,a)dwθ+∫abφ(w)Cw′(w,a)dw ×[φ(s)+∫sbφ(w)Cw′(w,s)dw]=C(t,s)−C(t,a)θ(∫sbφ(τ)Cτ′(τ,s)dτ+φ(s)) ×[1−∫abφ(w)Cw′(w,a)dwθ+∫abφ(w)Cw′(w,a)dw]=C(t,s)−C(t,a)∫sbφ(w)Cw′(w,s)dw+φ(s)θ+∫abφ(w)Cw′(w,a)dw,g(t)=C(t,a)θ(c−c∫abφ(s)Cs′(s,a)dsθ+∫abφ(s)Cs′(s,a)ds)=C(t,a)cθ+∫abφ(s)Cs′(s,a)ds,

and *C*(*t*, *s*) = 0, for *t* < *s*.

One has

(22) $$\begin{array}{c} x\left(t\right)=\int_{a}^{b}G\left(t,s\right)f\left(s\right)ds+g\left(t\right). \end{array}$$

Theorem 1 has been proven.

Theorem 1 demonstrates the importance to know exactly or approximately the Cauchy function *C*(*t*, *s*) of impulsive equations (1)–(4).

Substituting *c* = 0, *φ*(*s*) ≡ *γ*, *θ* = *γ* − 1 we obtain the Corollary.

**Corollary 2**

If generalized periodic problem (1)–(4), (7) is uniquely solvable, then its solution can be represented in the following form:

(23) $$\begin{array}{c} x\left(t\right)=\int_{a}^{b}W\left(t,s\right)f\left(s\right)ds, \end{array}$$

where the Green's function *W*(*t*, *s*) is as follows:

(24) $$\begin{array}{c} W\left(t,s\right)=C\left(t,s\right)-C\left(t,a\right)\frac{\gamma \int_{s}^{b}C_{w}^{\prime }\left(w,s\right)dw+\gamma }{\left(\gamma -1\right)+\gamma \int_{a}^{b}C_{w}^{\prime }\left(w,a\right)dw}. \end{array}$$

## 3. Auxiliary Results

Let us construct the Cauchy function *C*(*t*, *s*) for several simple equations.

Consider now the following auxiliary equation:

(25) $$\begin{array}{c} x^{\prime }\left(t\right)+p\left(t\right)x\left(t\right)=f\left(t\right). \end{array}$$

Let us denote the Cauchy function of the nonimpulsive equation (25) by *K*(*t*, *s*). It is known that *K*(*t*, *s*) = *e* ^−∫_*s*_^*t*^*p*(*ζ*)*dζ*^. For every *s* this function is absolutely continuous function with respect to *t*, and *K*(*t*, *s*) = 0 for *s* > *t*.

Using the fact that the Cauchy function *C*(*t*, *s*) for every fixed *s* as a function of *t* is a solution of

(26) $$\begin{array}{c} x^{\prime }\left(t\right)+p\left(t\right)x\left(t\right)=0,\;t\in \left[s,b\right] \end{array}$$

satisfying the condition *C*(*s*, *s*) = 1, we obtain the following theorem.

**Theorem 3**

The Cauchy function *C*(*t*, *s*) of (25) and (2) can be represented in the following form:

(27) C(t,s)=∑i=1k ∑j=0i−1 ∏r=j+1iβrK(t,s)[Hti(t)−Hti+1(t)]       ×[Htj(s)−Htj+1(s)] +∑i=0kH0(t−s)K(t,s)[Hti(t)−Hti+1(t)]     ×[Hti(s)−Hti+1(s)],

where *H*_*a*_(*t*), Heaviside function, is defined by the following equality:

(28) $$\begin{array}{c} H_{a}\left(t\right)=\{\begin{array}{cc} 1, & t\geq a,\\ 0, & t<a. \end{array} \end{array}$$

Now let us consider the following auxiliary equation:

(29) $$\begin{array}{c} x^{\prime }\left(t\right)=f\left(t\right),\;t\in \left[a,b\right],\\ x\left(t_{j}\right)=\beta_{j}x\left(t_{j}-0\right),\;j=1,\ldots ,k,\\ a=t_{0}<t_{1}<t_{2}<\cdots <t_{k}<t_{k+1}=b. \end{array}$$

**Figure 1:**
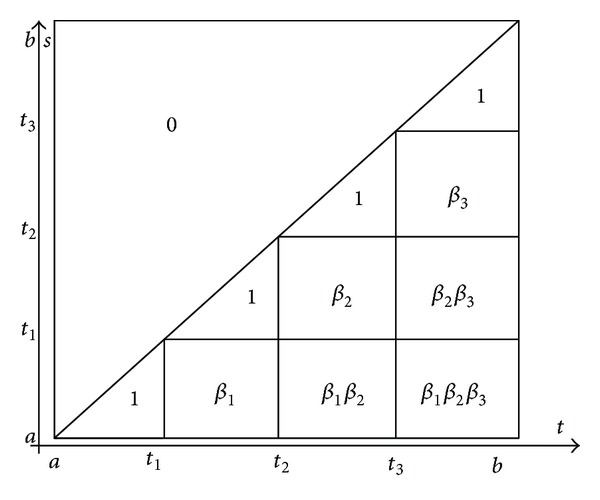


Figure 1 describes the Cauchy function *C*_0_(*t*, *s*) of the problem (29), (2), and (4) in the case *k* = 3.

In the case of *k* impulses we obtain the following theorem.

**Theorem 4**

The Cauchy function *C*_0_(*t*, *s*) of the problem (29), (2) and its derivatives has the following representations:

(30) C0(t,s)=∑i=1k ∑j=0i−1 ∏r=j+1iβr[Hti(t)−Hti+1(t)]      ×[Htj(s)−Htj+1(s)] +∑i=0kH0(t−s)[Hti(t)−Hti+1(t)]   ×[Hti(s)−Hti+1(s)],C0(t,a)=∑i=1k ∏r=1iβr[Hti(t)−Hti+1(t)] +H0(t−a)[Ht0(t)−Ht1(t)],(C0(t,s))t′=∑i=1k ∑j=0i−1 ∏r=j+1iβr[Htj(s)−Htj+1(s)]      ×[δti(t)−δti+1(t)] +∑i=0k[Hti(s)−Hti+1(s)]   ×(δ0(t−s)[Hti(t)−Hti+1(t)]    +H0(t−s)[δti(t)−δti+1(t)]),(C0(t,a))t′=∑i=1k∏r=1iβr[δti(t)−δti+1(t)]    +δ0(t−a)[Ht0(t)−Ht1(t)]    +H0(t−a)[δt0(t)−δt1(t)].

Denote by *G*_0_(*t*, *s*) Green's function of problem (29), (5), and by *W*_0_(*t*, *s*) Green's function of generalized periodic problem (29), (7).

Let us denote *I*(*φ*, *θ*, *β*) = *θ* + ∫_*a*_^*b*^ *φ*(*t*)(*C*_0_(*t*,*a*))_*t*_′*dt*.

Let *β* = *Col*{*β*_1_,…, *β*_*k*_}. The computation of integrals in formula (11) leads us to the following equality:

(31) I(φ,θ,β) =θ+∑i=1k−1∏r=1iβr     ×[φ(ti+)+φ(ti−)2−φ(ti+1+)+φ(ti+1−)2]     +β1β2⋯βk[φ(tk+)+φ(tk−)2−φ(tk+1−)2]     +φ(t0+)2−φ(t1+)+φ(t1−)2+φ(t0+)2.

Denoting *I*_1_(*φ*, *β*) = *φ*(*s*) + ∫_*s*_^*b*^ *φ*(*t*)(*C*_0_(*t*,*s*))_*t*_′*dt*, we obtain for *a* ≤ *s* < *t*_1_

(32) I1(φ,β)=φ(s) +∑i=1k−1∏r=1iβr   ×[φ(ti+)+φ(ti−)2−φ(ti+1+)+φ(ti+1−)2]   +β1β2⋯βk[φ(tk+)+φ(tk−)2−φ(tk+1−)2]   +φ(s+)2+H0(t0−s)φ(t0+)2−φ(t1+)+φ(t1−)2.

Denoting *I*_2_(*φ*, *β*) = *φ*(*s*) + ∫_*s*_^*b*^ *φ*(*t*)(*C*_0_(*t*,*s*))_*t*_′*dt*, we obtain for *t*_1_ ≤ *s* < *t*_2_

(33) I2(φ,β)=φ(s) +∑i=2k−1∏r=2iβr   ×[φ(ti+)+φ(ti−)2−φ(ti+1+)+φ(ti+1−)2]   +β2β3⋯βk[φ(tk+)+φ(tk−)2−φ(tk+1−)2]   +φ(s+)2+H0(t1−s)φ(t1+)2−φ(t2+)+φ(t2−)2⋮

Denoting *I*_*k*+1_(*φ*, *β*) = *φ*(*s*) + ∫_*s*_^*b*^ *φ*(*t*)(*C*_0_(*t*,*s*))_*t*_′*dt*, we obtain for *t*_*k*_ ≤ *s* < *b*

(34) Ik+1(φ,β)=φ(s)+φ(s+)2 +H0(tk−s)φ(tk+)2−  φ(tk+1−)2.  

Let us denote *J*(*β*, *γ*) = (*γ* − 1) + *γ*∫_*a*_^*b*^(*C*_0_(*t*,*a*))_*t*_′*dt*.

The computation of integrals in formula (24) leads us to the following equality:

(35) J(β,γ)=(γ−1) +  γ(∑i=1k∏r=1iβr[Hti(t)−Hti+1(t)]+1).

Denoting *J*_1_(*β*, *γ*) = *γ*∫_*s*_^*b*^(*C*_0_(*t*,*s*))_*t*_′*dt* + *γ*, we obtain for *a* ≤ *s* < *t*_1_

(36) J1(β,γ)=γ(∑i=1k∏r=1iβr[Hti(t)−Hti+1(t)]   +Ht0(s)+H0(t0−s)−1)+γ.

Denoting *J*_2_(*β*, *γ*) = *γ*∫_*s*_^*b*^(*C*_0_(*t*,*s*))_*t*_′*dt* + *γ*, we obtain for *t*_1_ ≤ *s* < *t*_2_

(37) J2(β,γ)=γ(∑i=2k∏r=1iβr[Hti(t)−Hti+1(t)]   +Ht1(s)+H0(t1−s))+γ⋮

Denoting *J*_*k*+1_(*β*) = *γ*∫_*s*_^*b*^(*C*_0_(*t*,*s*))_*t*_′*dt* + *γ*, we obtain for *t*_*k*_ ≤ *s* < *b*

(38) Jk+1(β)=γ(Htk(s)−Htk+1(s)+H0(tk−s)    −H0(tk+1−s))+γ=γ(Htk(s)+H0(tk−s))+γ.

**Corollary 5**

**Figure 2:**
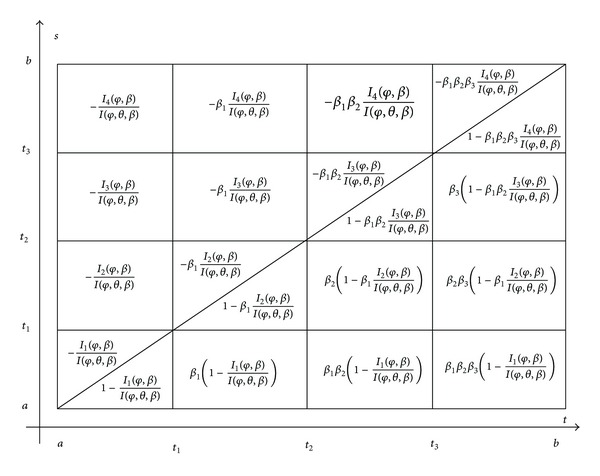


Let us assume that *I*(*φ*, *θ*, *β*)≠0; then Green's function *G*_0_(*t*, *s*) of problem (29), (5) in the case of three impulsive points (i.e., *k* = 3) is described by Figure 2.

**Corollary 6**

Let us assume that *J*(*β*, *γ*) ≠ 0; then Green's function *W*_0_(*t*, *s*) of generalized periodic problem (29), (7) in the case of three impulsive points (i.e. *k* = 3) is described by Figure 3.

## 4. Estimation of Solutions

Let us denote *β* = max⁡_1≤*i*≤*k*_⁡*β*_*i*_, *Ω* = (1/|*I*(*φ*, *θ*, *β*)|)|(1 − *β* ^*k*+1^)/(1 − *β*)|((2*k* + 1)/2)Δ where Δ = (max⁡_1≤*i*≤*k*+1_⁡|*t*_*i*_−*t*_*i*−1_|)^2^,

(39) Ω~=Ω[|θ|+||φ||(5+4∑i=1kβi)   +(max⁡1≤i≤k+1βi−1)||φ||(3+2∑i=1kβi)].

**Theorem 7**

Let 0 < *β*_*i*_, 1 ≤ *i* ≤ *k*,  *I*(*φ*, *θ*, *β*) ≠ 0 and

(40) $$\begin{array}{c} m\left(b-a\right)\underset{\underset{1\leq i\leq n}{s\in \left[a,b\right]}}{\text{ess}\;\sup}\left|p_{i}\left(s\right)\right|\tilde{\Omega }<1; \end{array}$$

then there exists a unique solution of problem (1)–(5) and this solution can be estimated as follows:

(41) ||x||≤Ω~(b−a)||f|||I(φ,θ,β)|+|c|max⁡[max⁡1≤j≤k⁡∏i=1jβi,1](1−Ω~)|I(φ,θ,β)|,

for every essentially bounded function *f* and real *c*.

**Proof**

By Theorem 1, problem (1)–(5) is equivalent to the integral equation:

(42) x(t)=∫abG0(t,s)ϕ(s)ds +  C0(t,a)cθ+∫abφ(s)(C0(s,a))s′ds=∫abG0(t,s)f(s)ds +  C0(t,a)cθ+∫abφ(s)(C0(s,a))s′ds −∫abG0(t,s)∑i=1mpi(s)x(s−τi(s))ds.

Let us denote *ψ*(*t*) = ∫_*a*_^*b*^ *G*_0_(*t*, *s*)*f*(*s*)*ds* + *C*_0_(*t*, *a*)(*c*/(*θ* + ∫_*a*_^*b*^ *φ*(*s*)(*C*_0_(*s*,*a*))_*s*_′*ds*)) = ∫_*a*_^*b*^ *G*_0_(*t*, *s*)*f*(*s*)*ds* + (*cC*_0_(*t*, *a*)/*I*(*φ*, *θ*, *β*)).

Define the operator *M* : *D*(*t*_1_,…, *t*_*k*_) → *D*(*t*_1_,…, *t*_*k*_) by the equality (*Mx*)(*t*) = −∫_*a*_^*b*^ *G*_0_(*t*, *s*)∑_*i*=1_^*m*^ *p*_*i*_(*s*)*x*(*s* − *τ*_*i*_(*s*))*ds*.

We can write equation which is equivalent to problem (1)–(5):

(43) $$\begin{array}{c} x\left(t\right)=\left(Mx\right)\left(t\right)+\psi \left(t\right). \end{array}$$

If ||*M*|| < 1, then there exists the unique solution of (1)–(5) which can be represented in the form: *x*(*t*) = (*I* − *M*)^−1^ *ψ*(*t*).

It is clear that

(44) ||Mx||≤||G0||∑i=1m∫ab|pi(s)||x(s−τi(s))|χ[a,b] ×(s−τi(s))ds≤||G0||m(b−a)ess sup⁡s∈[a,b]1≤i≤m|pi(s)|||x||,

where ||*x*|| = max⁡_*a*≤*t*≤*b*_⁡|*x*(*t*)|. Thus we have got the following condition for existence of unique solution of problem (1)–(5):

(45) $$\begin{array}{c} ||M||\leq ||G_{0}||m\left(b-a\right)\underset{\underset{1\leq i\leq n}{s\in \left[a,b\right]}}{\text{ess}\;\sup}\left|p_{i}\left(s\right)\right|<1. \end{array}$$

Denote

(46) $$\begin{array}{c} g\left(t\right)=\frac{cC_{0}\left(t,a\right)}{I\left(\varphi ,\theta ,\beta \right)}, \end{array}$$

where *C*_0_(*t*, *a*) can be written as

(47) $$\begin{array}{c} C_{0}\left(t,a\right)=\{\begin{array}{cc} 1, & a\leq t<t_{1},\\ \beta_{1}, & t_{1}\leq t<t_{2},\\ \beta_{1}\beta_{2}, & t_{2}\leq t<t_{3},\\ \vdots & \\ \beta_{1}\cdot \ldots \cdot \beta_{k}, & t_{k}\leq t<b. \end{array} \end{array}$$

It is clear that

(48) $$\begin{array}{c} ||g||\leq \frac{\left|c\right|\max\left[\max_{1\leq j\leq k}\prod_{i=1}^{j}\beta_{i},1\right]}{\left|I\left(\varphi ,\theta ,\beta \right)\right|}. \end{array}$$

Estimation of the solution of the problem (1)–(5) can be done as follows:

(49) |x(t)|≤||G0||∫ab|f(s)|ds+||g||+||Mx||   ⟹||x||   ≤||G0||(b−a)ess sup⁡t∈[a,b]|f(t)|    +||g||+||G0||m(b−a)ess sup⁡s∈[a,b]1≤i≤n|pi(s)|||x||   ⟹||x||(1−||G0||m(b−a)ess sup⁡s∈[a,b]1≤i≤n|pi(s)|)   ≤||G0||(b−a)||f||+||g||.

Let us denote

(50) $$\begin{array}{c} Q=||G_{0}||m\left(b-a\right)\underset{\underset{1\leq i\leq n}{s\in \left[a,b\right]}}{\text{ess}\;\sup}\left|p_{i}\left(s\right)\right|. \end{array}$$

Then the problem (1)–(5) has unique solution if

(51) $$\begin{array}{c} ||M||\leq Q<1. \end{array}$$

It is clear that

(52) $$\begin{array}{c} ||x||\leq \frac{||G_{0}||\left(b-a\right)||f||+||g||}{1-Q}. \end{array}$$

Estimating the integrals in formula (11), we get the following inequalities:

(53) |I(φ,θ,β)|≤|θ|+2||φ||+2||φ||∑i=1kβi=|θ|+||φ||(2+2∑i=1kβi),|I1(φ,β)|≤||φ||+2||φ||∑i=1kβi+2||φ||=3||φ||+2||φ||∑i=1kβi=||φ||(3+2∑i=1kβi),|I2(φ,β)|≤||φ||+2||φ||∑i=2kβi+2||φ||=3||φ||+2||φ||∑i=2kβi=||φ||(3+2∑i=2kβi),⋮|Ik+1(φ,β)|≤2||φ||.

Let us write *G*_0_ = *G*_1_ + *G*_2_, where *G*_1_ is upper triangular and *G*_2_ lower triangular parts of *G*_0_ and estimate ||*G*_0_||. One has

(54) ||G0||≤||G1||+||G2||≤Ω[max⁡1≤i≤k+1|Ii(φ,β)|   +max⁡1≤i≤k+1|I(φ,θ,β)−βi−1Ii(φ,β)|]≤Ω[max⁡1≤i≤k+1|Ii(φ,β)|+|I(φ,θ,β)|   +max⁡1≤i≤k+1|Ii(φ,β)|βi−1]=Ω[|I(φ,θ,β)|+max⁡1≤i≤k+1|Ii(φ,β)|(βi−1+1)].

From estimation of the integrals, we obtain

(55) ||G0||≤Ω[|θ|+||φ||(2+2∑i=1kβi)   +(1+max⁡1≤i≤k+1βi−1)||φ||(3+2∑i=1kβi)]≤Ω[|θ|+||φ||(5+4∑i=1kβi)   +(max⁡1≤i≤k+1βi−1)||φ||(3+2∑i=1kβi)]=Ω~.

By (50)

(56) Q≤m(b−a)ess sup⁡s∈[a,b]1≤i≤n|pi(s)|Ω ×[|θ|+||φ||(5+4∑i=1kβi)   +(max⁡1≤i≤k+1βi−1)||φ||(3+2∑i=1kβi)].

Substituting (56) into (51) and (52) we obtain

(57) ||M||≤m(b−a)ess sup⁡s∈[a,b]1≤i≤n|pi(s)|Ω ×[|θ|+||φ||(5+4∑i=1kβi)   +(max⁡1≤i≤k+1βi−1)||φ||(3+2∑i=1kβi)],

(58) ||x||≤(Ω~(b−a)||f||  +|c|max⁡[max⁡1≤j≤k⁡∏i=1jβi,1]|I(φ,θ,β)|)(1−Ω~)−1=(Ω~(b−a)||f|||I(φ,θ,β)|   +|c|max⁡[max⁡1≤j≤k∏i=1jβi,1])((1−Ω~)|I(φ,θ,β)|)−1.

Theorem 7 has been proven.

Let us denote   *Ω*_2_ = |1 − *β* ^*k*+1^|(2*k* + 1)Δ/2|1 − *β*||*J*(*β*, *γ*)|,

(59) Ω2~ =Ω2[|γ|(5+2∑i=1kβi)+max⁡1≤i≤k+1βi−1|γ|(3+∑i=1kβi)].

**Theorem 8**

Let 0 < *β*_*i*_, 1 ≤ *i* ≤ *k*,  *J*(*β*, *γ*) ≠ 0 and

(60) $$\begin{array}{c} \tilde{\Omega_{2}}m\left(b-a\right)\underset{\underset{1\leq i\leq m}{s\in \left[a,b\right]}}{\text{ ess }\;\sup}\left|p_{i}\left(s\right)\right|<1; \end{array}$$

then there exists a unique solution of the generalized periodic problem (1)–(4), (7) and this solution can be estimated as follows:

(61) $$\begin{array}{c} ||x||\leq \frac{\;\;\tilde{\Omega_{2}}\left(b-a\right)||f||}{1-\tilde{\Omega_{2}}m\left(b-a\right)\underset{\underset{1\leq i\leq m}{s\in \left[a,b\right]}}{\text{ ess }\;\sup}\left|p_{i}\left(s\right)\right|} \end{array}$$

for every essentially bounded function *f*.

**Proof**

By Corollary 2, problem (1)–(4), (7) is equivalent to the following integral equation:

(62) x(t)=∫abW0(t,s)ϕ(s)ds=∫abW0(t,s)f(s)ds −∫abW0(t,s)∑i=1mpi(s)x(s−τi(s))ds.

Let us denote *ψ*(*t*) = ∫_*a*_^*b*^ *W*_0_(*t*, *s*)*f*(*s*)*ds*.

Define the operator *T* : *D*(*t*_1_,…, *t*_*k*_) → *D*(*t*_1_,…, *t*_*k*_) by the equality (*Tx*)(*t*) = −∫_*a*_^*b*^ *W*_0_(*t*, *s*)∑_*i*=1_^*m*^ *p*_*i*_(*s*)*x*(*s* − *τ*_*i*_(*s*))*ds*.

We can write equation which is equivalent to problem (1)–(4), (7) as follows:

(63) $$\begin{array}{c} x\left(t\right)=\left(Tx\right)\left(t\right)+\psi \left(t\right). \end{array}$$

If ||*T*|| < 1, then there exists the unique solution of (1)–(4), (7) which can be represented in the form: *x*(*t*) = (*I* − *M*)^−1^ *ψ*(*t*).

It is clear that

(64) ||Tx||≤||W0||∑i=1m∫ab|pi(s)||x(s−τi(s))|χ[a,b]        ×(s−τi(s))ds≤||W0||m(b−a)ess sup⁡s∈[a,b]1≤i≤m|pi(s)|||x||,

where ||*x*|| = max⁡_*a*≤*t*≤*b*_⁡|*x*(*t*)|. Thus we have got the following condition for existence of unique solution of problem (1)–(4), (7):

(65) $$\begin{array}{c} ||T||\leq ||W_{0}||m\left(b-a\right)\underset{\underset{1\leq i\leq n}{s\in \left[a,b\right]}}{\text{ess}\;\sup}\left|p_{i}\left(s\right)\right|<1. \end{array}$$

Estimation of the solution of the problem (1)–(4), (7) can be done as follows:

(66) |x(t)|≤||W0||∫ab|f(s)|ds+||Tx||   ⟹||x||   ≤||W0||(b−a)ess sup⁡t∈[a,b]|f(t)|    +||W0||m(b−a)ess sup⁡s∈[a,b]1≤i≤m|pi(s)|||x||   ⟹||x||(1−||W0||m(b−a)ess sup⁡s∈[a,b]1≤i≤m|pi(s)|)   ≤||W0||(b−a)||f||.

Let us denote

(67) $$\begin{array}{c} Q=||W_{0}||m\left(b-a\right)\underset{\underset{1\leq i\leq m}{s\in \left[a,b\right]}}{\text{ess}\;\sup}\left|p_{i}\left(s\right)\right|. \end{array}$$

Then the problem (1)–(4), (7) has unique solution if

(68) $$\begin{array}{c} ||T||\leq Q<1. \end{array}$$

It is clear that

(69) $$\begin{array}{c} ||x||\leq \frac{||W_{0}||\left(b-a\right)||f||}{1-Q}. \end{array}$$

Estimating the integrals in formula (24), we get the following inequalities:

(70) $$\begin{array}{c} \left|J\left(\beta ,\gamma \right)\right|\leq \left|\gamma \right|+\left|\gamma \right|\left(1+\sum_{i=1}^{k}\beta^{i}\right)=\left|\gamma \right|\left(2+\sum_{i=1}^{k}\beta^{i}\right),\\ \left|J_{1}\left(\beta ,\gamma \right)\right|\leq \left|\gamma \right|\left(\sum_{i=1}^{k}\beta^{i}+1\right)+\left|\gamma \right|\leq \left|\gamma \right|\left(3+\sum_{i=1}^{k}\beta^{i}\right),\\ \;\;\;\;\;\;\left|J_{2}\left(\beta ,\gamma \right)\right|\leq \left|\gamma \right|\left(\sum_{i=2}^{k}\beta^{i}+2\right)+\left|\gamma \right|\leq \left|\gamma \right|\left(3+\sum_{i=2}^{k}\beta^{i}\right),\\ \vdots \\ \;\;\;\;\;\;\left|J_{k+1}\left(\beta \right)\right|=3\left|\gamma \right|,\\ \underset{1\leq i\leq k+1}{\sup}\left|J_{i}\left(\beta ,\gamma \right)\right|\leq \left|\gamma \right|\left(3+\sum_{i=1}^{k}\beta^{i}\right). \end{array}$$

Let us write *W*_0_ = *W*_1_ + *W*_2_, where *W*_1_ is upper triangular and *W*_2_, lower triangular parts, and estimate ||*W*_0_||:
(71)
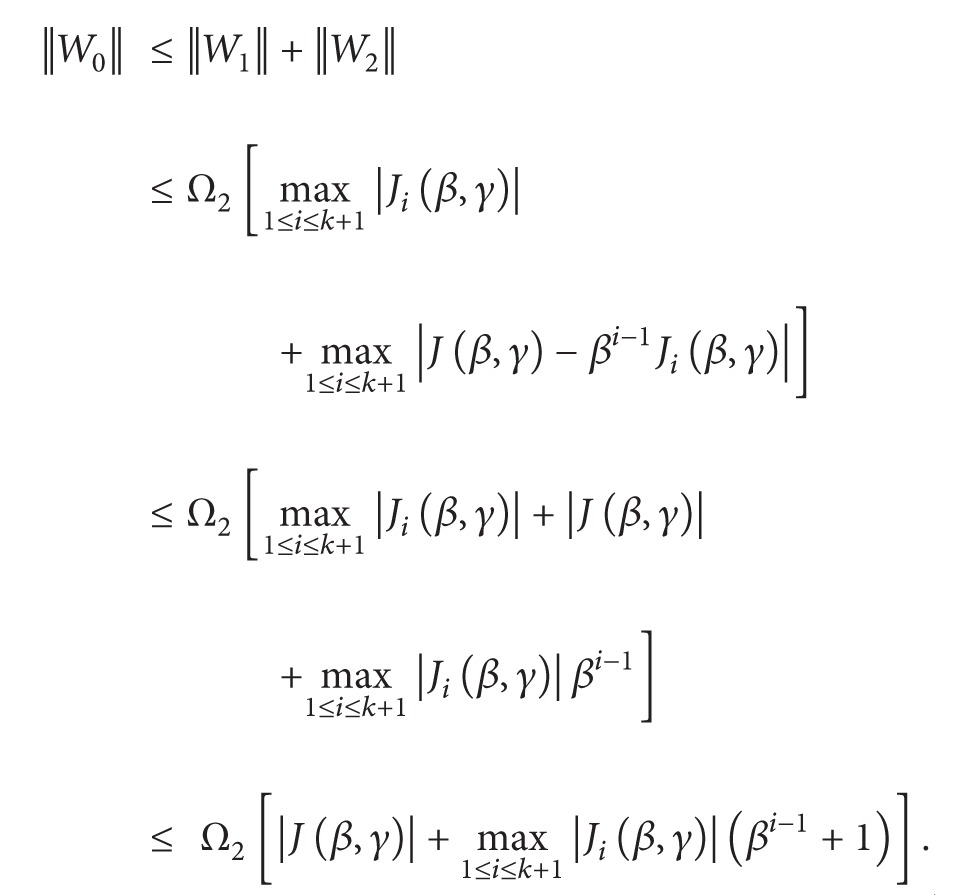

We obtain
(72)
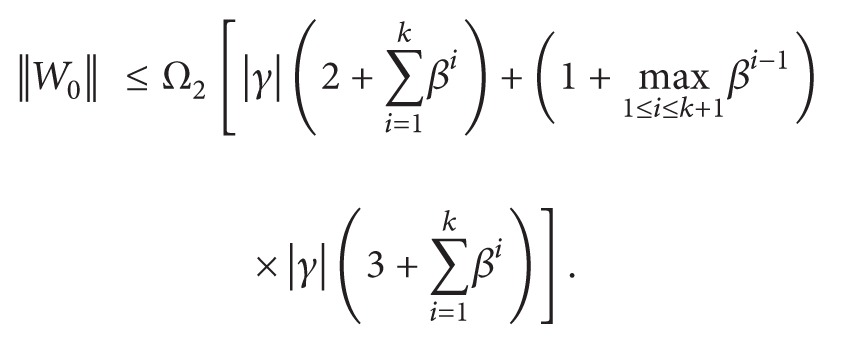

It is clear that

(73) ||T||≤Q≤Ω2[|γ|(5+2∑i=1kβi)   +max⁡1≤i≤k+1βi−1|γ|(3+∑i=1kβi)] ×m(b−a)ess sup⁡s∈[a,b]1≤i≤m|pi(s)|=Ω2~m(b−a)sup⁡s∈[a,b]1≤i≤m|pi(s)|.

We obtain the following solution estimation:

(74) $$\begin{array}{c} ||x||\leq \frac{\;\;\tilde{\Omega_{2}}\left(b-a\right)||f||}{1-\tilde{\Omega_{2}}m\left(b-a\right)\underset{\underset{1\leq i\leq m}{s\in \left[a,b\right]}}{\text{ess}\;\sup}\left|p_{i}\left(s\right)\right|}. \end{array}$$

Theorem 8 has been proven.

## 5. Sign Constancy of Green's Functions

The proofs of the following two assertions follow from the construction of Green's functions.

**Theorem 9**

Let

(75) 0<βi, 1≤i≤k,I(φ,θ,β)·Ij(φ,θ,β)<0, j=1,…,k+1;

then Green's function *G*_0_(*t*, *s*) of problem (29), (5) is positive for *t*, *s* ∈ [*a*, *b*].

**Theorem 10**

Let

(76) 0<βi, 1≤i≤k,  I1(φ,θ,β)I(φ,θ,β)>1,Ij(φ,θ,β)I(φ,θ,β)>1∏i=1j−1βi 2≤j≤k+1;

then Green's function *G*_0_(*t*, *s*) of problem (29), (5) is negative for *t*, *s* ∈ [*a*, *b*].

Estimation of Green's function (11) leads us to the assertion.

**Theorem 11**

Let conditions (40) and (75) be fulfilled, *p*_*i*_(*t*) ≤ 0 for *i* = 1,…, *m*; then problem (1)–(5) is unique solvable and its Green's function *G*(*t*, *s*) is positive for *t*, *s* ∈ [*a*, *b*].

**Proof**

Without losing generality we assume that *c* = 0.

Solution of problem (1)–(5) can be represented in the following form:

(77) $$\begin{array}{c} x\left(t\right)=\left(Mx\right)\left(t\right)+\psi \left(t\right), \end{array}$$

where

(78) $$\begin{array}{c} \left(Mx\right)\left(t\right)=-\int_{a}^{b}G_{0}\left(t,s\right)\sum_{i=1}^{m}p_{i}\left(s\right)x\left(s-\tau_{i}\left(s\right)\right)ds,\\ \psi \left(t\right)=\int_{a}^{b}G_{0}\left(t,s\right)f\left(s\right)ds.\; \end{array}$$

Using Theorem 9, we obtain that the operator *M* is positive.

The conditions of Theorem 7 imply that ||*M*|| < 1.

Now it is clear that
(79)
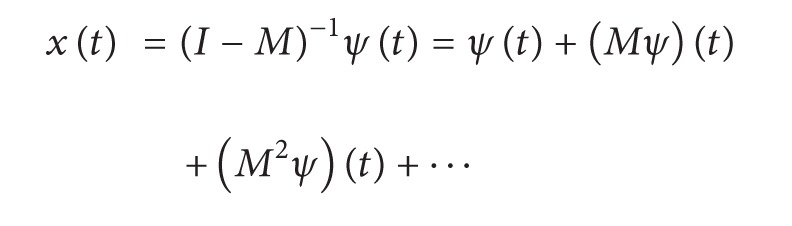

It follows from the positivity of the operator *M* that for every nonnegative *f* we get according to (78) *ψ*(*t*) ≥ 0 and *x*(*t*) ≥ *ψ*(*t*) ≥ 0.

It is clear that

(80) 0≤x(t)−ψ(t)=∫abG(t,s)f(s)ds−∫abG0(t,s)f(s)ds=∫ab[G(t,s)−G0(t,s)]f(s)ds.

From nonnegativity of *x*(*t*) for every nonnegative *f*(*t*), we obtain that *G*(*t*, *s*) ≥ *G*_0_(*t*, *s*) ≥ 0.

Theorem 11 has been proven.

**Theorem 12**

Let conditions (40) and (76) be fulfilled, *p*_*i*_(*t*) ≥ 0 for *i* = 1,…, *m* then problem (1)–(5) is unique solvable and its Green's function *G*(*t*, *s*) is negative for *t*, *s* ∈ [*a*, *b*].

We prove this assertion analogously to the proof of Theorem 11.

The proofs of the following two assertions follow from the construction of Green's functions.

**Theorem 13**

Let

(81) 0<βi, 1≤i≤k,  J1(β,γ)J(β,γ)>1,Jj(β,γ)J(β,γ)>1∏i=1j−1βi 2≤j≤k+1;

then Green's function of problem (29), (7) *W*_0_(*t*, *s*) is negative for *t*, *s* ∈ [*a*, *b*].

**Theorem 14**

Let

(82) $$\begin{array}{c} 0<\beta_{i},\;1\leq i\leq k,\;\;\underset{1\leq i\leq k+1}{\max}J\left(\beta ,\gamma \right)\cdot J_{i}\left(\beta ,\gamma \right)<0; \end{array}$$

then Green's function of problem (29), (7) *W*_0_(*t*, *s*) is positive for *t*, *s* ∈ [*a*, *b*].

The proof of the following two theorems can be obtained analogously from the proof of Theorem 11.

**Theorem 15**

Let conditions (60) and (82) be fulfilled, *p*_*i*_(*t*) ≤ 0 for *i* = 1,…, *m*; then problem (1)–(4), (7) is unique solvable and its Green's function *W*(*t*, *s*) is positive for *t*, *s* ∈ [*a*, *b*].

**Theorem 16**

Let conditions (60) and (81) be fulfilled, *p*_*i*_(*t*) ≥ 0 for *i* = 1,…, *m*; then problem (1)–(4), (7) is unique solvable and its Green's function *W*(*t*, *s*) is negative for *t*, *s* ∈ [*a*, *b*].

## Figures and Tables

**Figure 3:**